# Antifungal Biocontrol in Sustainable Crop Protection: Microbial Lipopeptides, Polyketides, and Plant-Derived Agents

**DOI:** 10.3390/jof12010022

**Published:** 2025-12-27

**Authors:** Nadya Armenova, Lidia Tsigoriyna, Alexander Arsov, Stefan Stefanov, Kaloyan Petrov, Wanmeng Mu, Wenli Zhang, Penka Petrova

**Affiliations:** 1 Institute of Chemical Engineering, Bulgarian Academy of Sciences, 1113 Sofia, Bulgaria; n.armenova@iche.bas.bg (N.A.); lidia@iche.bas.bg (L.T.); s.stefanov@iche.bas.bg (S.S.); kkpetrov@iche.bas.bg (K.P.); 2 Institute of Microbiology, Bulgarian Academy of Sciences, 1113 Sofia, Bulgaria; al.arsov@microbio.bas.bg; 3 State Key Laboratory of Food Science and Technology, Jiangnan University, Wuxi 214122, China; wmmu@jiangnan.edu.cn (W.M.); wenlizhang@jiangnan.edu.cn (W.Z.)

**Keywords:** antifungal lipopeptides, polyketides, fungal phytopathogens, biological control, synthetic biology

## Abstract

Fungal phytopathogens cause significant global crop losses and remain a constant obstacle to sustainable food production. Biological control has become a vital alternative to synthetic fungicides, supported by the wide variety of antifungal molecules produced by bacteria, fungi, yeasts, and plants. This review consolidates current knowledge on the main classes of microbial secondary metabolites—particularly cyclic lipopeptides and polyketides from *Bacillus*, *Pseudomonas*, *Streptomyces*, *Trichoderma*, and related genera. It emphasizes their structural diversity, biosynthetic pathways, regulatory networks, and antifungal mechanisms. These molecules, including iturins, fengycins, surfactins, syringomycins, candicidins, amphotericin analogs, peptaibols, and epipolythiodioxopiperazines, target fungal membranes, mitochondria, cell walls, and signaling systems, offering broad activity against damaging pathogens such as *Fusarium*, *Botrytis*, *Magnaporthe*, *Colletotrichum*, *Phytophthora*, and *Rhizoctonia*. The plant-derived antifungal metabolites include essential volatile compounds that complement microbial agents and are increasingly important in eco-friendly crop protection. Recent progress in genomics, metabolic engineering, and synthetic biology has accelerated strain improvement and the discovery of new bioactive compounds. At the same time, global market analyses indicate rapid growth in microbial biofungicides driven by regulatory changes and consumer demand.

## 1. Introduction

The rapidly increasing global population drives demand for agricultural and food products, necessitating the development of innovative and effective strategies for plant protection. According to estimates by the Food and Agriculture Organization of the United Nations (FAO), global food production needs to increase by approximately 70% to meet the nutritional requirements of a projected world population of nearly 9.6 billion people by 2050 [1,2]. The challenge for modern agriculture, therefore, is to achieve high crop yields and food quality while maintaining sustainability and ecological safety in production systems [3]. Modern research emphasizes the development of integrated plant disease management strategies that combine biological control agents, resistant crop varieties, and precise agricultural technologies. These efforts also aim to reduce chemical inputs, prevent the development of pathogen resistance, and minimize ecosystem contamination, while ensuring a high food supply.

Fungal pathogens can significantly reduce agricultural productivity (up to 30%), causing numerous plant diseases that lead to substantial yield declines and quality losses in crops [4,5]. They can reproduce rapidly and adapt quickly, resulting in resistance to commonly used fungicides. Moreover, infections can occur during both cultivation and postharvest stages, affecting nearly every part of the food production process [6].

Staple crops of high economic and agricultural importance, such as rice, wheat, maize, potato, and soybean, are continually threatened by various fungal diseases, posing serious challenges to global food production and food security [7,8]. Mildew, rust, and mold remain among the most destructive threats to global agriculture, making fungicides the most widely used plant protection chemicals. Usually, these are synthetic compounds, like imidazoles and dithiocarbamates. Although effective, these substances can leave harmful residues in food crops, promote the development of resistant fungal strains, and pose risks to human health and the environment [9]. They are also not permitted in organic farming because of the potential for food contamination [10].

As a result, the search for biological alternatives—biofungicides—has concentrated on all possible microbial producers of antifungal compounds. Among the most studied are soil-dwelling *Bacillus species*, such as *B. amyloliquefaciens*, *B. velezensis*, *B. subtilis*, *B. nakamurai*, *B. siamensis*, and *B. licheniformis*, which all show strong antagonistic activity, mainly through the production of secondary metabolites, especially lipopeptides and polyketides [11,12,13]. Many other microbial species exhibit similar activity but are less well researched, including members of the genus *Streptomyces* and fungal antagonists such as *Trichoderma harzianum*.

This review highlights recent progress in the microbial-based production of lipopeptides and polyketides, identifies leading microbial producers of these antifungal compounds, and provides a brief examination of their synthesis and mechanisms of action. It also discusses emerging genetic engineering methods to enhance the production of these essential bioactive metabolites.

## 2. Overview and Economic Assessment of the Biofungicide Market

### 2.1. Biofungicide Production Annual Growth Rate

**Figure 1:**
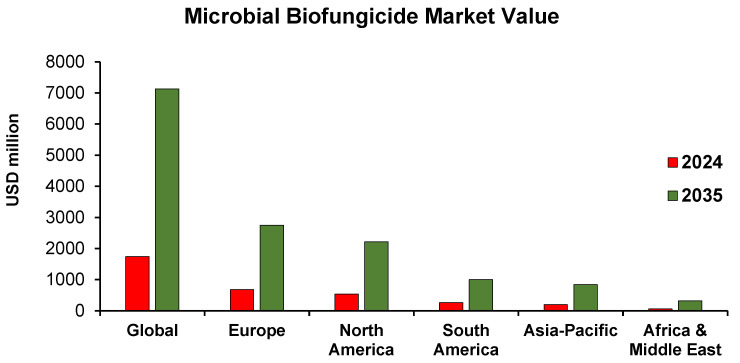
Microbial biofungicide market value forecast for 2035 compared to 2024 [14].

The biofungicide market is experiencing steady growth. Several reports estimate the Compound Annual Growth Rate (CAGR) to range from a cautious 0.6% to a more optimistic 14.5%, with no reports indicating a negative CAGR. Most market analyses forecast an average CAGR of 11% to 14% over the next decade [13,14,15]. A trend shows that the more optimistic CAGR estimates tend to start with a smaller initial market size; for example, a report with a CAGR of 14.5% predicts a 2024 market size of USD 885.60 million [14], while another with an 8.61% CAGR estimates a 2025 market size of USD 1722.62 million [15]. Although the forecasts vary, all suggest the market will grow significantly over the next 10 years, reaching a global value of USD 2150–8315 million, up from its current USD 2930 million, corresponding to 1350 kilotons of biofungicides used in 2024. Regarding classification, the market can be segmented and analyzed based on type or volume. Based on the first criterion, biofungicides can be categorized into microbial and botanical types, with microbial-based biofungicides accounting for 62–67% of the market and plant extract-based biofungicides accounting for the remaining 33–38%. A report estimates that the microbial biofungicide market will grow by 415% over the next 11 years. It is currently valued at USD 1040 million and is expected to reach USD 4330 million by 2035. The botanical segment, meanwhile, is projected to grow by 400%, increasing from USD 700 million to USD 2800 million. In terms of volume, in 2024, the global biofungicide market is valued at USD 1740 million, with Europe (USD 680 million) and North America (USD 540 million) being the largest regional markets, followed by South America (USD 260 million), Asia-Pacific (USD 200 million), and Africa and the Middle East (USD 60 million). By 2035, the market is expected to grow significantly to USD 7130 million worldwide, with Europe (USD 2750 million) and North America (USD 2220 million) remaining the main contributors [15]. All other regions are also expected to see considerable expansion. The regional market value segmentation, along with the 2035 forecast, is illustrated in Figure 1.

### 2.2. Global Biofungicide Use

**Figure 2:**
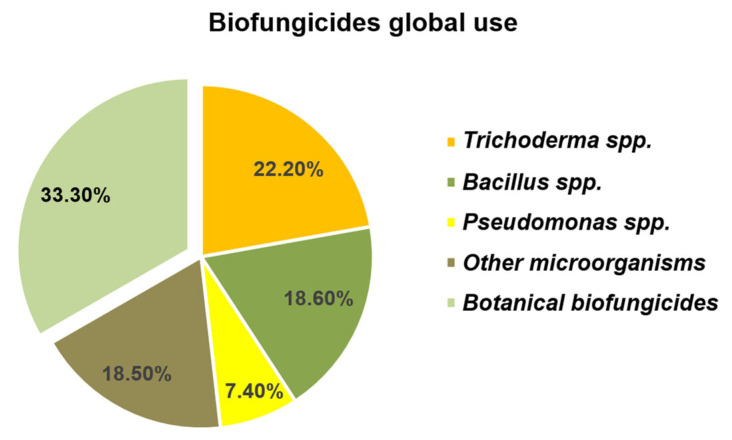
Global use of biofungicides in 2024 and the main microbial genera involved in the production [16].

The global use of biofungicides in 2024 reached 1350 kt, accounting for 35% of the worldwide fungicide market by volume—a 25% increase since 2020. It also shows a 30% rise in biofungicide use in organic farming from 2022 to 2024, covering 4.5 million hectares. The report states that out of 900 kt of microbial biofungicides used in 2024 (which account for 67% of the total), the most common products include *Trichoderma* (~300 kt), *Bacillus* (~250 kt), and *Pseudomonas* (~100 kt), with the remaining 250 kt utilizing various other microorganisms (Figure 2).

The Asia-Pacific region was the leading consumer, with 350 kt during this period. The global grains and fruits sectors used 280 and 250 kt, respectively. The worldwide market for microbial biofungicides is growing rapidly and is forecasted to reach USD 1.2 billion in 2025. These products are becoming the dominant segment of biological crop protection, accounting for approximately 70% of the market. Most of the demand originates from the United States, where microbial agents are widely used on grains and vegetables, and from China, which extensively applies them in fruit and vegetable production. India’s expanding organic farming sector, Brazil’s large-scale soybean and maize cultivation, and Germany’s national programs supporting sustainable agriculture further propel this growth.

Plant-derived biofungicides are also expanding. In 2024, global production reached about 450,000 tons, mainly comprising neem-based products and essential oils. These plant-derived agents are mainly applied to fruits and vegetables, with particularly high use in Mediterranean orchards. The economic value of this segment is projected to exceed 500 million USD in 2025. India and China, both major producers of botanical pesticides, are expected to lead this growth, followed by the United States through its strong organic food sector, Brazil with its soybean production, and France through initiatives promoting environmentally friendly farming practices.

Another way to classify biofungicides is by formulation: liquid or dry. The former includes ready-to-use products, such as suspensions, which account for 58% of total liquid biofungicides, along with emulsifiable concentrates and other liquid formulations. The latter encompasses dry and wettable powders, granules, and similar forms [16]. Both have their advantages and disadvantages. For example, liquid biofungicides are easier to apply, distribute evenly, and are quickly absorbed by plants; they also work well with most spraying equipment used on large farms, with 88% of manufacturers certifying compatibility. Additionally, liquid formulations better maintain microbial strain viability under field conditions than dry forms, though their shelf life is shorter [3]. Currently, dry formulations account for a larger market segment, although not by a significant margin [13]. In contrast, the market split 53/47 between liquid and dry options [16].

Conversely, the liquid suspension segment is expected to grow the fastest due to its versatility, efficiency, and ease of use across both conventional and organic farming. The main advantages of dry suspensions include their stability without special storage conditions, compact size, and suitability for specific applications. For instance, granules are often used for soil application targeting root pathogens because they remain effective over long periods [14]. Regarding market distribution by region for 2024, the reports are consistent. The Asia-Pacific region remains the global leader, with biofungicide usage of approximately 500 kt. Europe and North America appear to have relatively similar market sizes, at 350 and 400 kt, respectively. In comparison, the still-developing Middle East and Africa market shows a respectable biofungicide usage of about 100 kt. The EU’s efforts to ease regulations by implementing changes under the EU Biopesticides Directive enabled mutual approval of active ingredients across the 27 member states, resulting in a 22% reduction in the number of registration processes within the EU.

In 2024, 38% of organic fruits grown in Europe used biofungicides, up from 24% in 2021. Public–private partnerships invested USD 37 million in the research and development (R&D) of region-specific microbial strains, boosting production in Europe by 29% [16]. While each report segments the biofungicide market differently, primarily based on application, some general figures for 2024 can be identified: the fruits and vegetables market consumed between 430 and 520 kt, and grains and oilseeds (and cereals) between 290 and 830 kt, while 300 kt was used for other applications such as ornamentals, turf, forestry, etc.

### 2.3. Global and Emerging Manufacturers of Biofungicides

Major corporations dominate a significant portion of the market. Bayer CropScience AG controlled 16–18% of the market and produced 215,000 tons of biofungicide in 2024. BASF SE held a 12–15% stake and manufactured 185 million liters of liquid biofungicide and 160,000 tons of dry biofungicide. Other key players include Monsanto Company, DOW Chemical Company, Corteva Agriscience, Syngenta AG, Marrone Bio Innovations Inc., Novozymes, Isagro SPA, Valent Biosciences Corporation, Gowan Group, AgraQuest, and FMC Corporation. These companies invest significant capital in their research and development departments, often collaborating with or supporting various smaller research startups, emerging companies, and scientific institutions [13]. About 120 biofungicide trials were registered with national agricultural authorities in 2023, while 35 new commercial products were launched in 2024 (up from 27 in 2023), including 22 microbial suspensions and 13 plant extracts. Fourteen co-formulations of biofungicides, biostimulants, and micronutrients were introduced to the market in 2024, providing plant protection and growth enhancement. Microbial strains accounted for 60% of new registrations in 2024 (up from 45% in 2021), with 145 registered biofungicide strains used commercially in over 50 countries [16].

## 3. Fungal Phytopathogens and Their Microbial Antagonists

Biofungicides are effective against a broad spectrum of phytopathogenic fungi, including those responsible for major plant diseases such as smut, rust, gray mold, powdery mildew, downy mildew, early blight, late blight, anthracnose, wilt, root rot, stem rot, rice blast, apple scab, black rot, citrus black spot, damping-off, charcoal rot, and various canker diseases. Among the numerous fungi that infect plants, several groups stand out as particularly destructive pathogens because of the significant agricultural losses they cause.

### 3.1. Top Pathogenic Fungi Causing Significant Agricultural Losses

According to Dean et al., (2012), who reviewed this issue a decade ago, the most devastating phytopathogenic fungi belong to *Puccinia* spp., *Fusarium* (*F. graminearum* and *F. oxysporum*), *Colletotrichum*, *Magnaporthe oryzae*, *Botrytis cinerea*, *Blumeria graminis*, *Mycosphaerella graminicola*, *Melampsora lini*, *Ustilago maydis*, *Phakopsora pachyrhizi*, and *Rhizoctonia solani* [17]. Today, the list may be expanded to include *Ph. infestans*, *Alternaria* spp., *Neocosmospora* spp., and various *Aspergillus* species [11,18,19,20]. Beyond their immediate destructive effects, *Fusarium*, *Alternaria*, and *Aspergillus* produce stable mycotoxins that can persist in food even after processing, posing serious health risks to humans and animals, including carcinogenic, hepatotoxic, nephrotoxic, and immunosuppressive effects [21]. *Phytophthora infestans*, an oomycete responsible for late blight, is one of the most damaging pathogens affecting *Solanaceae* crops, particularly potatoes and tomatoes [17].

The rice blast fungus *M. oryzae*, an ascomycete from the family *Magnaporthaceae*, infects cultivated rice, causing blighting lesions on leaves, stems, and heads. It is now found in more than 80 countries worldwide and also infects other cereals such as millet and wheat [22]. *Botrytis cinerea* (gray mold fungus) is a necrotrophic ascomycete (class *Leotiomycetes*, family *Sclerotiniaceae*) with an extensive host range. It attacks fruits and vegetables (such as strawberries, grapes, and tomatoes), as well as ornamentals and greenhouse crops, producing gray, fuzzy mold on decaying plant tissue, and is a major postharvest problem in cool, humid regions [23].

The genus *Puccinia* includes obligate basidiomycete rust fungi (order *Uredinales*, family *Pucciniaceae*) that cause rust diseases on cereals and grasses. For example, *P. graminis* (wheat stem rust) infects dozens of wheat, barley, oats, and rye species, producing brick-red uredinia on stems and leaves. *Puccinia* species often have complex life cycles with alternate hosts and cause rust outbreaks globally. In cereals, stem rust (*P. graminis*), leaf rust (*P. triticina*), and stripe rust (*P. striiformis*) are among the most detrimental diseases of wheat and barley worldwide [24]. *Fusarium graminearum* (teleomorph *Gibberella zeae*) is an ascomycete in the order *Hypocreales* (family *Nectriaceae*). It causes *Fusarium* head blight, also known as scab, on small grains. This pathogen infects wheat and barley heads, causing stalk and ear rot on maize. These fungal outbreaks are problematic in North America, Europe, and Asia. The soil-borne *F. oxysporum* (an ascomycete) causes vascular wilts by invading roots and clogging the plant’s xylem. It appears in several host-specific forms: *F. oxysporum* sp. *cubense* infects bananas (causing Panama disease), *F. oxysporum* sp. *lycopersici* infects tomatoes, and *F. oxysporum* sp. *pisi* infects peas [25]. Similarly, *Blumeria graminis* (cereal powdery mildew) also has subspecies that attack specific plants: *B. graminis* sp. *tritici* on wheat and sp. *hordei* on barley. It is an obligate biotrophic ascomycete (order *Erysiphales*, family *Erysiphaceae*) appearing as white powdery lesions on leaves and stems, being one of the top fungal diseases of wheat, causing significant yield losses [26].

*Mycosphaerella graminicola*, now known as *Zymoseptoria tritici*, is an ascomycete (order *Mycosphaerellales*, family *Mycosphaerellaceae*). It causes Septoria leaf blotch on wheat, infecting the foliage and producing chlorotic spots and necrotic blotches. This disease is a major concern in Europe, Africa, and other wheat-growing regions. *Z. tritici* is hemibiotrophic and reproduces sexually on infected residues. It primarily infects wheat (and some wild grasses) and is a key pathogen in modern wheat fields [27].

*Colletotrichum* spp. (order *Glomerellales*, family *Glomerellaceae*), causes anthracnose and fruit-rot diseases on many crops. Members of this genus infect fruits (strawberries, citrus, mango, olives, blueberries, coffee, etc.), vegetables (pepper, tomato), and even some cereals and grasses [28].

*Ustilago maydis* is a basidiomycete (order *Ustilaginales*, family *Ustilaginaceae*) that causes “common smut” of maize. It infects ears, tassels, and stems of corn, producing large tumor-like galls filled with black teliospores, and is totally biotrophic. Infected kernels swell into grayish-white galls that release masses of spores [29].

### 3.2. Microbial Strains Applicable in the Biological Control of Phytopathogenic Fungi

#### 3.2.1. Bacteria

Microbial fungicides are mainly based on bacterial strains and their secondary metabolites, as well as on fungi and yeasts with antifungal properties. The bacterial component primarily consists of plant-growth-promoting rhizobacteria (PGPR), soil microorganisms, epiphytes, and mycorrhizal fungi. PGPR are considered environmentally safe and effective because they produce diverse antifungal compounds, including antimicrobial peptides, lipopeptides, polyketides, and siderophores. Well-known PGPR include *Bacillus subtilis*, *B. amyloliquefaciens*, *B. licheniformis*, *B. cereus*, *Pseudomonas fluorescens*, *P. syringae*, *Rhizobium* spp., and others [30]. These bacteria suppress a broad spectrum of soil-borne fungal pathogens, including *Fusarium*, *Rhizoctonia solani*, *Macrophomina*, *Alternaria*, *Penicillium*, *Cladosporium*, and *Humicola* species [31].

Several *Bacillus* strains have demonstrated vigorous biocontrol activity under both field and postharvest conditions. *B. subtilis*, *B. amyloliquefaciens*, and *Ps. stutzeri* effectively suppress *Phytophthora capsici* in cucumber roots [32], while *B. subtilis* QST 713 protects tomato fruits from *Penicillium* spp. and *Rhizopus stolonifer* during storage [33]. *B. amyloliquefaciens* strains significantly reduce *Fusarium* wilt caused by *F. oxysporum* ssp. *lycopersici* [34] and inhibit citrus green mold (*P. digitatum*) through the production of macrolactin, bacillaene, iturins, fengycin, and surfactin. Other *Bacillus* isolates suppress *Botrytis cinerea*, reducing gray mold and powdery mildew in strawberry and cucumber crops [12].

Specific antifungal metabolites play a crucial role in these interactions. Bacillomycin D, produced by *B. amyloliquefaciens* FZB42, inhibits *F. graminearum*, while fengycin from *B. subtilis* BS155 damages the membrane integrity of *Magnaporthe grisea*, causing oxidative stress and hyphal death [35]. Iturins and bacillomycin F produced by *B. siamensis* strains exhibit strong activity against *Colletotrichum*, *R. solani*, and *M. grisea*, and the secretion of chitinase and β-1,3-glucanase further improves antifungal effectiveness against *Fusarium* wilt in tomato [36]. *B. velezensis* SDTB038 controls *Fusarium* crown and root rot through the combined action of multiple bioactive metabolites, including bacillaene, bacilysin, difficidin, fengycin, macrolactin, and surfactin [37].

*Pseudomonas* species are also key biocontrol agents, especially against root-rot and vascular pathogens. *P. piscium* ZJU60 inhibits *F. graminearum* by reducing virulence and mycotoxin production through phenazine-1-carboxamide secretion [38], while *P. aeruginosa* manages anthracnose in chili peppers and triggers systemic resistance in the host plant [39]. However, resistance issues can develop even with biofungicides: studies on the product Howler EVO, derived from *P. chlororaphis*, showed cross-resistance with the synthetic fungicide fludioxonil in *B. cinerea*, highlighting the importance of resistance management strategies [40].

In addition to *Bacillus* and *Pseudomonas*, soil-derived *Streptomyces* species demonstrate strong antifungal potential. Many isolates inhibit pathogens such as *Fusarium* spp., *R. solani*, *B. cinerea*, *Alternaria*, *Colletotrichum*, *Ganoderma boninense*, and *Phytophthora* spp. Optimizing fermentation conditions and using genetic engineering have further enhanced antifungal activity in selected strains, for example, by increasing biomass production or removing regulatory genes that negatively impact secondary metabolite biosynthesis [41]. Endophytic *Streptomyces* isolates have also shown promising results in greenhouse trials, reducing disease incidence while also promoting plant growth [42]. Overall, PGPR that combine biocontrol activity with plant growth promotion are beneficial for sustainable agriculture, as they enhance plant resilience, combat a wide variety of phytopathogens, and decrease dependence on chemical fungicides.

#### 3.2.2. Yeast and Fungal Strains

Other microorganisms frequently applied as biofungicides include yeasts and filamentous fungi such as *Candida*, *Coniothyrium*, *Ampelomyces*, *Gliocladium*, and *Trichoderma* spp., which are characterized by rapid growth, adaptability, and high specificity toward target phytopathogens. Yeasts have been extensively studied for postharvest disease control, particularly in citrus. *Candida oleophila* and *Hanseniaspora anomala* effectively suppress *P. digitatum*, *P. italicum*, and *Geotrichum candidum*, with protection levels increasing at higher antagonist concentrations and longer pre-inoculation intervals, reaching up to 100% disease suppression under optimized conditions [43]. Screening of microbial isolates from citrus fruit surfaces identified yeasts (*C. oleophila*, *Debaryomyces hansenii*) and *Bacillus* species (*B. amyloliquefaciens*, *B. pumilus*, *B. subtilis*) as effective antagonists against citrus green and blue molds. These microorganisms reduced disease incidence during cold storage without negatively affecting fruit quality, acting through multiple mechanisms, including biofilm formation, lipopeptide production, lytic enzymes, and volatile compounds [44].

Among fungal biofungicides, *Trichoderma* spp. represent the most commercially successful group, accounting for more than half of all registered biological disease-control formulations worldwide [45]. Numerous *Trichoderma* strains inhibit a broad range of phytopathogens, including *Fusarium*, *R. solani*, *Pythium*, *Sclerotium rolfsii*, *Penicillium*, *Aspergillus*, *Alternaria*, *Phytophthora*, *Pyricularia*, *Botrytis*, and *Gaeumannomyces* [46]. Their biocontrol activity is primarily mediated by the production of cell wall–degrading enzymes such as chitinases, glucanases, and proteases. Species including *T. harzianum*, *T. viride*, *T. atroviride*, *T. hamatum*, and *T. asperellum* are widely commercialized.

Beyond direct antagonism, *Trichoderma* strains also enhance plant defense responses. Endophytic colonization by *T. asperellum* ICC012 and *T. gamsii* ICC080 significantly reduced *Fusarium* head blight in wheat and upregulated defense-related genes, demonstrating both protective and growth-promoting effects [46].

Other fungal genera, including *Penicillium*, *Gliocladium*, *Aspergillus*, *Saccharomyces*, and *Chaetomium*, also exhibit antagonistic activities through parasitism and secondary metabolite production against pathogens [47]. In addition, arbuscular mycorrhizal fungi contribute indirectly to disease suppression by enhancing nutrient uptake, inducing systemic resistance, and improving plant vigor. For example, *Funneliformis mosseae* in combination with *Sinorhizobium medicae* suppresses *F. oxysporum* in alfalfa [48].

Antagonistic yeasts are also effective against root and collar diseases, primarily through competition for nutrients and space, rapid surface colonization, and the secretion of lytic enzymes. Several yeast and fungal species exhibit direct mycoparasitism, including *Rhodotorula* spp. against *Monilinia*, *Tuberculina maxima* against rust fungi, and *Tilletiopsis* spp. against cucumber powdery mildew, underscoring the diversity of fungal and yeast-based mechanisms available for biological disease control [49].

### 3.3. Plant Secondary Metabolites

Modern disease management strategies in agriculture include the use of environmentally friendly plant extracts. Plants can produce numerous secondary metabolites. These compounds are usually classified by biosynthetic origin into groups such as terpenes and terpenoids, polysaccharides, phenolic compounds, sulfur-containing phytoalexins, nitrogen-containing alkaloids, flavonoids, and various hydrocarbons, all of which have antifungal properties and help defend the plant against pathogens [50]. Plant-based biofungicides work through different mechanisms of action. These include blocking germ tube growth and spore formation, disrupting DNA replication and protein synthesis, damaging hyphal and mycelial structures, and reducing the production of toxic metabolites and mycotoxins by pathogenic fungi [51]. Additionally, plants produce essential oils and volatile compounds that also have strong antifungal properties, inhibiting the growth of various pathogenic fungi, such as those derived from thyme (*Thymus vulgaris*, Thyme Guard^®^, Agro Research International LLC, Sorrento, FL, USA), oregano (*Origanum vulgare*), rosemary (*Rosmarinus officinalis*), mint (*Mentha* spp.), basil (*Ocimum basilicum*), giant knotweed (*Reynoutria sachalinensis*, Regalia^®^, ProFarm Group Inc., Davis, CA, USA), and citrus species, which disrupt fungal cell membranes and suppress phytopathogens, including *Botrytis*, *Fusarium*, *Alternaria*, and *Penicillium* spp. [52].

Examples of the antifungal effectiveness of different plant extracts against these and other fungal species include studies by Latinović et al. [53] and Sabithira and Udayakumar [54]. The first authors reported that methanolic extracts from *Porella platyphylla*, *Cinclidotus fontinaloides*, and *Anomodon viticulosus* significantly inhibited the mycelial growth of *B. cinerea*, highlighting the potential of bryophyte-derived metabolites as promising natural sources of biofungicidal compounds. Conversely, the second study emphasizes the high inhibition of *A. niger*, *A. flavus*, *A. terreus*, *T. viride*, and *F. solani* by the leaves’ and stems’ extracts of *Marsilea minuta*.

Various studies have further examined the antifungal properties of angiosperm extracts. For example, ethanolic leaf extracts of *Ipomoea batatas* L. (sweet potato) significantly slowed the growth of *Fusarium* species [55]. Cruz et al. reported that the hydroethanolic extract of nutmeg, *Myristica fragrans*, contained essential oils, phenolic compounds, and alkaloids that showed vigorous antifungal activity against *F. oxysporum*, *Botrytis cinerea*, *Colletotrichum acutatum*, *Diplodia corticola*, and *Ph. cinnamomi*, mainly by disrupting ergosterol biosynthesis in fungal cell membranes [56]. In another study, the same group found that *Curcuma longa* hydroethanolic extract exhibited both antifungal and antioomycete effects against *C. acutatum*, *B. cinerea*, *P. cinnamomi*, *F. culmorum*, and *D. corticola* due to its high content of bisabolene sesquiterpenoids [57]. Sobhy et al. [58] tested the methanolic extract of *Cinnamomum camphora* for antifungal activity against *Alternaria alternata*, *F. solani*, and *F. oxysporum*. At 4000 µg/mL, the extract reduced mycelial growth by up to 60%. HPLC analysis identified catechin and gallic acid as the most abundant phenolics, which likely contributed to its antifungal effect. In a study by Salas-Gómez et al., polyphenol extracts from mistletoe plants growing on three different tree species (mesquite *Prosopis glandulosa* Torr, cedar *Cedrus* Trew, and oak *Quercus* L.) were tested for antifungal activity against several tomato pathogens—including *Alternaria alternata*, *F. oxysporum*, and *R. solani*. The extracts, containing flavones, anthocyanins, and luteolin, showed significant inhibition of pathogen growth [59]. Wei et al. demonstrated that phenolic acids extracted from rice straw can activate resistance in tomato plants against *F. oxysporum*. Their results showed that these phenolic extracts damage the fungal cell membrane, increasing permeability and causing cytoplasmic leakage. This disruption ultimately prevents spore germination and hyphal growth of the pathogen, highlighting the potential of rice-straw-derived phenolic acids as natural biofungicide agents [60].

In a study by Al-Askar et al., the methanolic extract of the whole plant of *Eryngium campestre* L. was tested for antimicrobial activity against fungal and bacterial pathogens isolated from symptomatic potato plants. The extract, analyzed by HPLC, contained several polyphenolic compounds, including benzoic acid, catechol, quercetin, vanillic acid, resveratrol, naringenin, and quinol. The target pathogens included *R. solani*, *F. oxysporum*, *F. solani*, *Dickeya solani*, and *Pectobacterium carotovorum*. Antimicrobial assays showed that the extract inhibited fungal growth in a concentration-dependent manner, with the highest activity against *F. solani* and *F. oxysporum*. Similarly, bacterial pathogens were inhibited in a dose-dependent manner, with *D. solani* exhibiting the most extraordinary sensitivity [61].

In a study by García-Ramírez et al. [62], the antifungal activity of cinnamon essential oil (*Cinnamomum zeylanicum* J. Presl), neem oil (*Azadirachta indica* A. Juss), and black sapote (*Diospyros digyna*) fruit extract was evaluated against postharvest fungal pathogens. The extracts were tested in vitro against *F. oxysporum*, *F. solani*, *Goetrichum* sp., and *Ph. capsici*. The results demonstrated that cinnamon oil exhibited a strong fungicidal effect at all tested concentrations. Neem oil exhibited notable antifungal activity, particularly at 400 ppm, where it reduced the mycelial growth of *F. solani* and *F. oxysporum* by 42.3% and 27.8%, respectively. At 350 ppm, it inhibited *P. capsici* and *Goetrichum* sp. by 53.3% and 20.9%, respectively. The black sapote extract exhibited moderate inhibitory effects, reducing the growth of all tested fungi by 21.9–28.6% at 400 ppm. These findings suggest that applying plant-derived extracts, particularly cinnamon and neem oils, can effectively reduce or prevent postharvest fungal infections in chayote fruit, offering a natural, eco-friendly alternative to synthetic fungicides. In a study by Ordóñez et al., the methanolic extracts of *Pernettya prostrata* and *Rubus roseus* Schott were tested for their antibacterial and antifungal effects against *Ph. infestans* and *Neopestalotiopsis javaensis*, which cause banana bacterial wilt, tomato late blight, and avocado scab, respectively. The results showed that both plant extracts exhibited inhibitory activity against *P. infestans*, with minimum inhibitory concentrations (MICs) of 31.25 mg/mL [63]. In a study by Hernández-Álvarez et al., the antifungal potential of ethanolic root extracts from both wild and cultivated specimens of *Argyranthemum frutescens* was evaluated in vitro against *B. cinerea*, *F. oxysporum*, and *Alternaria alternata*. The analysis identified several polyacetylenes with potent antifungal activity, including over 90% growth inhibition of *B. cinerea* at 0.05 mg/mL. Additionally, capillinol and capillin showed greater activity than commercial fungicides Fosbel-Plus and Azoxystrobin against *F. oxysporum*.

The summary of microbial and plant biocontrol agents, their targets, and their mode of action is presented in Table 1.

## 4. Microbial Antifungal Metabolites and Molecules—Classes and Targets

The growing understanding of antifungal organisms, their bioactive metabolites, and the underlying molecular mechanisms can lay a strong foundation for creating next-generation biofungicides. Advances in genomics and metabolic engineering now allow for the enhancement of microbial strains to boost stability, efficacy, and activity in diverse environmental conditions. Continued interdisciplinary research that links microbiology, plant pathology, and molecular genetics will be crucial for transforming laboratory findings into durable, field-ready biofungicide products.

### 4.1. Cyclic Lipopeptides Produced by Bacillus spp.

**Figure 3:**
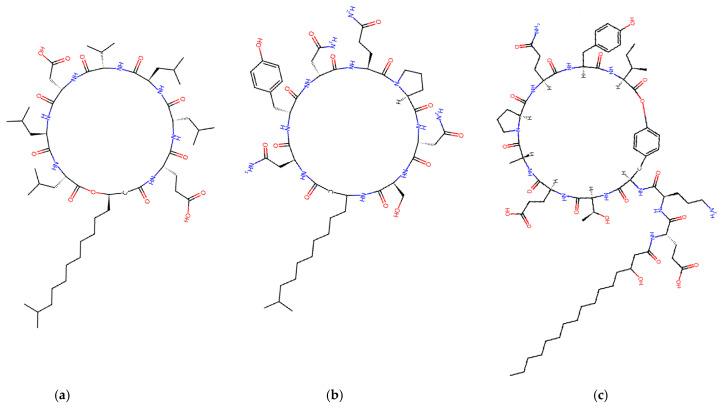
Structural formulas of (**a**) Surfactin; (**b**) Iturin A; (**c**) Fengycin. Available online at https://pubchem.ncbi.nlm.nih.gov/ (accessed on 12 November 2025).

Spore-forming *Bacillus* species are among the most common producers of biofungicides, mainly because they can synthesize amphiphilic cyclic lipopeptides using nonribosomal peptide synthetases (NRPSs). The three prominent families, whose structures are shown in Figure 3, are: (i) surfactins: primarily known for their surfactant and biofilm-disrupting properties; (ii) iturins: potent membrane-active molecules that bind to sterols in fungal membranes, creating pores and causing leakage of potassium ions and metabolites; and (iii) fengycins: particularly effective against filamentous fungi destabilizing lipid bilayers and inhibiting phospholipase activity. The simultaneous production of these families within a single strain is observed in many commercial *Bacillus* products, providing broad antifungal coverage and reducing the risk of resistance.

#### 4.1.1. Surfactins

Although surfactins are less directly toxic to fungi, they enhance dispersion and work synergistically with other lipopeptides. Surfactin consists of a peptide loop of seven amino acids (L-glutamate, L-leucine, D-leucine, L-valine, L-aspartate, D-leucine, and L-leucine), linked to β-hydroxy fatty acid chains of varying lengths (C12–C16). These compounds exhibit significant structural diversity and are classified into the esperin, lichenysin, pumilacidin, and surfactin groups [65]. Their amphiphilic structure imparts notable physicochemical properties [66,67] and various biological activities [68]. The antibacterial activity of surfactin is closely linked to its interactions with microbial biofilms. It disturbs membrane integrity by destabilizing lipid bilayers and forming transient pores or channels that permit the passage of intracellular components, including proteins, nucleic acids, and potassium ions, leading to rapid cell death [69]. Among various applications, surfactin is used for food preservation [70] and as a biofungicide in agriculture [71,72].

Xiao et al. [73] examined the mechanisms of surfactin’s antifungal action against *B. cinerea*. The results showed that surfactin can significantly inhibit pathogen growth by disrupting fungal membranes in a dose-dependent manner. Another example is purified surfactin produced by *B. subtilis* SF1, which strongly inhibits the growth of *F. foetens* mycelium at 20 μg/μL, causing hyphal deformation, leakage of cellular contents, changes in protein expression, and accumulation of reduced glutathione [74]. Surfactin has also been shown to enhance the biological activities of other lipopeptides against plant pathogens [75]. A mixture of surfactin and fengycin, derived from *B. subtilis*, is highly effective against the causative agent of grapevine downy mildew, *Plasmopara viticola*. The supernatant directly inhibits this oomycete and also stimulates plant defense [76].

#### 4.1.2. Iturins

The iturin family is characterized by a seven–amino acid peptide ring attached to a β-amino fatty acid, whose alkyl chain can be either linear or branched. In nature, these lipopeptides typically exist as a mixture of several closely related variants, mainly differing in the length of their β-hydroxy fatty acid chains (C13–C18) and whether they have n- or iso-configured chains. It includes several variants such as iturin (A, C, D, E), bacillomycin (D, F, L), bacillopeptin, and mycosubtilins.

The antifungal activity of iturin results from its interaction with the cytoplasmic membrane of target cells, increasing potassium ion permeability and forming ion-conducting pores in fungal cell membranes [77]. Primarily produced by *B. subtilis* and *B. amyloliquefaciens* strains, iturins demonstrate strong antifungal activity against various fungal phytopathogens, including *F. graminearum* [78], *F. oxysporum* [79], *A. niger* [80], *R. solani* [81], *Ph. infestans* [82], and *B. cinerea* [83].

#### 4.1.3. Fengycins

Fengycin is a cyclic lipopeptide composed of a β-hydroxy fatty acid chain (C14–C17) attached to a cyclic decapeptide core. The peptide chain includes both L- and D-amino acids, with the specific amino acid makeup and chain length varying among fengycin isomers. Members of the fengycin family include types A and B [84], fengycin S [85], fengycin C [86], and plipastatins A and B [87,88,89,90].

Compared with surfactin and iturin A, fengycin shows a more pronounced antagonistic effect on filamentous fungi by disrupting the cell membrane, leading to permeability and structural changes that cause leakage of cellular contents and cell death [91,92].

Fengycin, mainly produced by *B. amyloliquefaciens* and *B. subtilis*, promotes plant growth and effectively combats various fungi, including *C. gloeosporioides* [92], *Magnaporthe grisea* [93], *Plasmodiophora brassicae* [94], *Botryosphaeria dothidea* [95]; *F. solani* ssp. *radicicola* [96], *F. graminearum* [97], *F. oxysporum,* and notably, its subspecies *physali* [98,99].

### 4.2. Cyclic Lipopeptides Produced by Brevibacillus spp.

**Figure 4:**
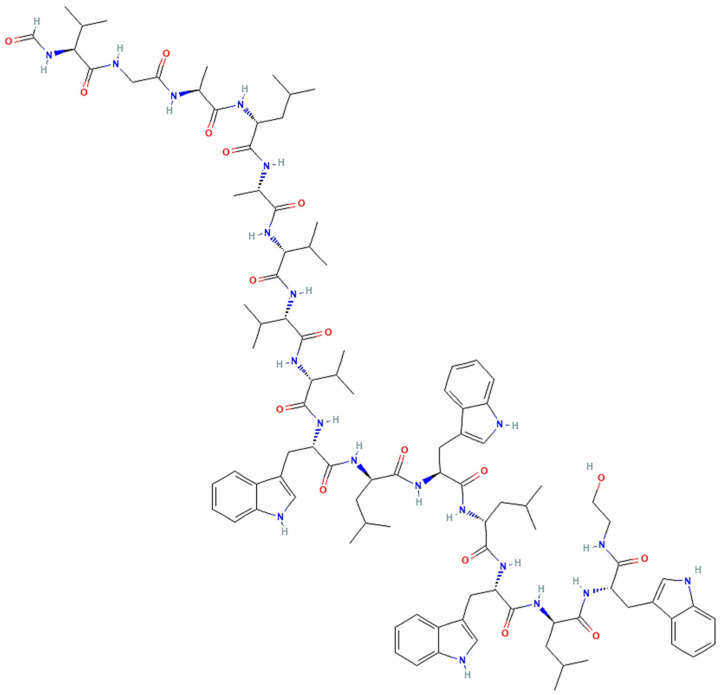
Structure formula of Gramicidin A. Available online at https://pubchem.ncbi.nlm.nih.gov/ (accessed on 19 December 2025).

*Brevibacillus* spp. can produce a wide variety of bioactive peptides with antibacterial and antifungal activity and is used as a biocontrol agent against plant diseases [100,101]. In *Brevibacillus*, antimicrobial peptides belong to diverse structural groups: bacteriocin, lipopeptide, cyclic peptide, and polyketides, and are synthesized through either ribosomal or nonribosomal biosynthetic pathways. The nonribosomal linear peptides include tostadin, gramicidin A–C (Figure 4), and edeine, whereas the nonribosomal cyclic decapeptides comprise gramicidin S, tyrocidine A–C, laterocidin, and loloatins A–D [102,103].

Gramicidin exerts its antimicrobial effect by forming transmembrane channels that disrupt the cell’s ion balance, ultimately causing cell death. It inserts into the lipid bilayer to form a pore, allowing sodium and potassium ions to pass freely and collapsing the electrochemical gradient essential for cellular function. This ion imbalance impairs metabolism and can lead to membrane lysis. Additionally, gramicidin A may trigger the formation of hydroxyl radicals [104].

Brevistin is a cyclic lipopeptide consisting of 11 amino acids and a fatty acid chain, first isolated in 1975 from the bacterium *B. brevis* 342-14 [105]. Brevicillin, a novel lanthipeptide from the genus *Brevibacillus*, exhibits antimicrobial, antifungal, and antiviral activities [106]. The lipopeptide brevilaterin B is a promising agent for agricultural biocontrol and postharvest storage. The research provided insights into the ability of brevilaterin B to control *F. oxysporum* and *P. chrysogenum* [107]. *B. laterosporus* was used as a potent biocontrol agent with both insecticidal and antifungal properties [100].

### 4.3. Antifungal Secondary Metabolites Produced by Actinomycetes

Actinomycetes, especially *Streptomyces* spp., are prolific producers of antifungal polyketides (e.g., nystatin-like macrolides, candicidins), and hybrid products of polyketide synthases and NRPS.

Polyene macrolides are a well-known class of over 200 antifungal agents produced mainly by *Streptomyces* species, of which amphotericin B, candicidin, nystatin, and natamycin are the most commonly used “gold standard” in the treatment of fungal infections [108].

These macrocyclic polyketides, synthesized by Type I modular polyketide synthases (PKSs), consist of a macrolactone ring, conjugated double bonds (usually 3–7), containing one or more sugar residues [109]. These secondary metabolites often target ergosterol biosynthesis or bind directly to fungal cell membranes, thereby affecting permeability and fluidity, disrupting homeostasis, and inducing oxidative damage [110].

**Figure 5:**
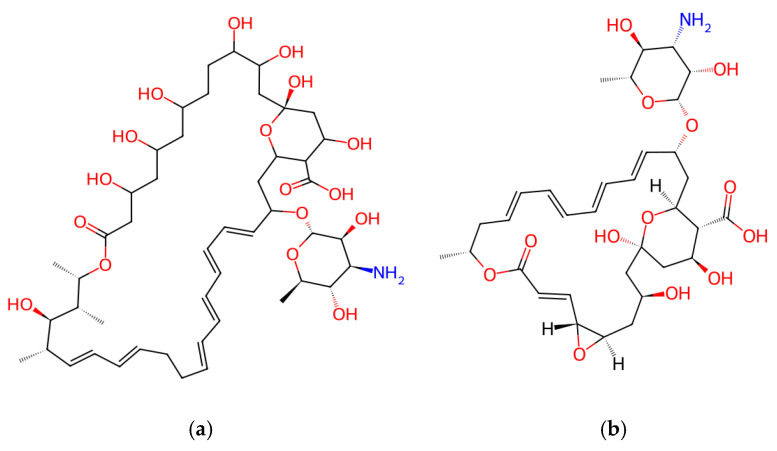
Antifungal secondary metabolites of *Streptomyces* spp.: (**a**) Nystatin; (**b**) Natamycin. Available online at https://pubchem.ncbi.nlm.nih.gov/ (accessed on 12 November 2025).

*S. nodosus* naturally produces amphotericin B. It was first introduced in 1958 and has remained a key treatment option for severe fungal infections for over fifty years [111]. A representative example of the polyene class is natamycin (pimaricin) with the structure shown in Figure 5, originally isolated from *S. natalensis*. It is synthesized by several species of *Streptomyces*, including *S. gilvosporeus*, *S. chattanoogensis*, and *S. lydicus*. Due to its antifungal activity, natamycin is widely used as a natural food preservative (E235) [112].

In agriculture, candicidin is widely used as a biocontrol agent, particularly effective against many plant pathogenic fungi and yeasts (*B. cinerea*, *R. solani*, *Fusarium* spp., *Alternaria* spp.). It also plays a role in seed treatment, soil application, and foliar sprays [113].

### 4.4. Cyclic Lipopeptides Produced by Pseudomonas spp.

Lipopeptides produced by *Pseudomonas* are classified as lipodepsipeptides with potent antifungal activities against a broad spectrum of fungi, including human pathogens [114]. They are characterized by the presence of ester bonds, which replace one or more typical amide bonds in the peptide chain. This feature explains the use of “depsi” in their name and sets them apart from ordinary cyclic peptides, which contain only amide bonds. Their structure typically combines an N-terminal fatty acyl group with a macrocyclic peptide ring that may include multiple ester bonds, along with various non-proteinogenic amino acids characteristic of nonribosomal assembly. This arrangement, pairing a hydrophobic tail with a cyclic depsipeptide core, explains their strong surface activity and antifungal properties [115]. Biosynthesis occurs through modular NRPS enzymes that activate and incorporate specific amino acids, form ester linkages, load a fatty acid starter unit derived from primary metabolism, and ultimately cyclize and release the final product via a thioesterase-mediated step.

**Figure 6:**
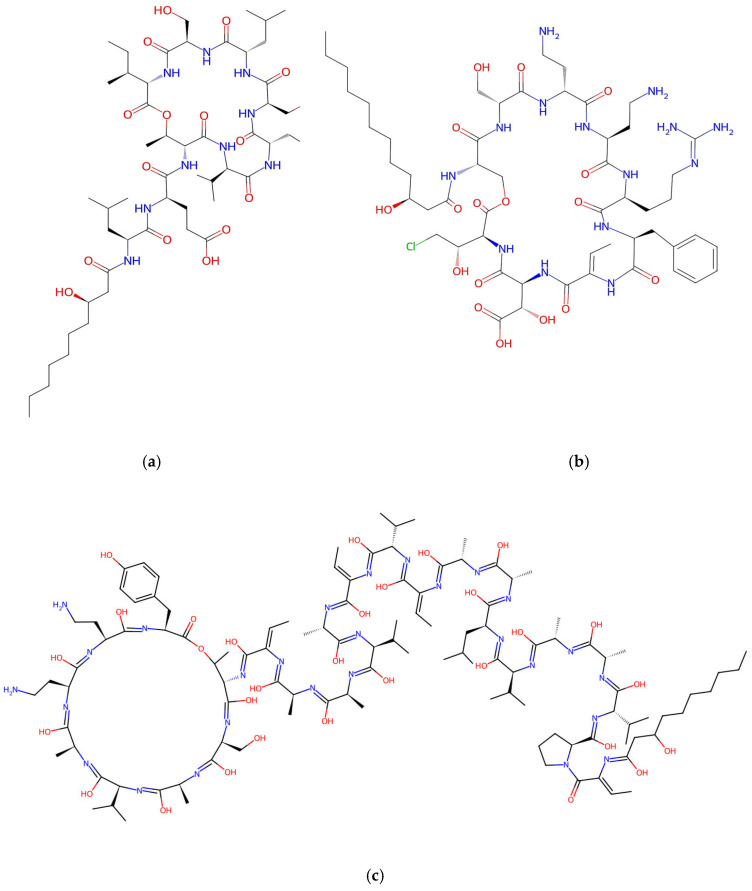
Antifungal secondary metabolites of *Pseudomonas* spp.: (**a**) Viscosin; (**b**) Syringomycin; (**c**) Syringopeptin. Available online at https://pubchem.ncbi.nlm.nih.gov/ (accessed on 22 November 2025).

Lipodepsipeptides from *Pseudomonas* are broadly divided into four prominent structural families: viscosin, syringomycin, tolaasin, and amphisin [116], of which the syringomycin group includes the cyclic lipodepsinonapeptides characterized by a polar peptide head and a 3-hydroxy fatty acid tail; these consist of four subclasses: syringomycins (Figure 6b), syringostatins, syringotoxins, and pseudomycins [117,118,119,120]. Their production relies on the conserved biosynthetic and export genes *syrB* and *syrD* [121], and related phytotoxic lipodepsipeptides, such as the 22-residue syringopeptins, also bearing a 3-hydroxy fatty acid chain, have been isolated from *Ps. syringae* pv. *syringae*.

Members of the syringomycin and syringopeptin class are pore-forming cytotoxins that act by promoting passive transmembrane ion flux [122], exhibit antibiotic activities against filamentous fungi and yeast, and function as a virulence determinant in the plant–pathogen interaction [123,124].

The cyclic lipodepsinonapeptide syringomycin E produced by *Ps. syringae* is employed as a biocontrol agent against fungal diseases on postharvest lemons and oranges [125]. Plant growth-promoting *Ps. putida* strain 267 produces two NRPSs involved in the production of the CLPs putisolvin I and II (homologues of PsoA and PsoB), which show zoosporicidal activities and inhibit the growth of the fungal pathogens *Ph. capsici*, *B. cinerea*, and *R. solani* [126].

A summary of the antifungal secondary metabolites and the genetic basis of their production by the bacterial strains is presented in Table 2.

### 4.5. Polyketide-Derived Antifungal Metabolites Produced by Fungi

Fungal polyketides are structurally diverse natural products with potent antimicrobial activity, including polyene macrolides, strobilurins, griseofulvin, lovastatin, and azaphilones with applications in commercial drugs and agricultural fungicides.

Strobilurins are a class of fungal secondary metabolites produced through polyketide biosynthesis. They are naturally found in small wood-inhabiting basidiomycetes, including *Strobilurus* species, where they serve as chemical defenses. Their structures are derived from a polyketide backbone formed by successive condensations of acetate units, followed by specific enzymatic modifications that produce the characteristic active part. This structural feature allows strobilurins to inhibit mitochondrial respiration by blocking electron transport at complex III. The combination of aromatic rings, oxygen-containing groups, and various substituents gives these molecules a unique chemical profile and broad biological activity.

Strobilurins are an important group of broad-spectrum agricultural fungicides, including strobilurin A, azoxystrobin, kresoxim-methyl, and pyraclostrobin, which fight fungal diseases caused by ascomycetes, basidiomycetes, and oomycetes. They protect a variety of crops—such as vegetables, rice, coffee, wheat, and vineyards [146]. Their mechanism of action involves blocking mitochondrial respiration by specifically binding to the Qo site (outer quinol oxidation site) of cytochrome b, preventing electron transfer between cytochrome b and cytochrome c1, which inhibits NADH oxidation and ATP production at the mitochondrial membrane, ultimately causing cell death [147].

Another polyketide, griseofulvin, is an antifungal metabolite obtained from cultures of *P. griseofulvum*. It selectively inhibits microtubule assembly in phytopathogenic fungi and is also used in human and veterinary medicine [148]. In agriculture, griseofulvin serves as a crop protectant to prevent fungal colonization and infection, support plant growth, and boost their resistance to diseases [149,150].

*Trichoderma* spp. are a well-known source of antibiotics, plant growth promoters, enzymes, and commercial biocontrol agents [37]. Extensive research has focused on the production of a wide variety of metabolites with unique chemical structures and notable biological activities, including polyketides [151], terpenoids [152], steroids [153], and peptides. For example, peptaibols produced by *Trichoderma* spp. are linear nonribosomal peptides, typically composed of 7 to 20 amino acid residues and often containing non-proteinogenic amino acids such as α-aminoisobutyric acid. They are rich in an unusual amino acid, 2-aminoisobutyric acid (Aib), and contain an N-terminal acyl group, such as acetate, along with a C-terminal amino alcohol [154]. These peptides are synthesized by nonribosomal peptide synthetases (NRPSs, Table 3) [155].

Peptaibols are usually divided into three groups based on their amino acid chain length: long-chain (18–20 amino acids), short-chain (11–16 residues), and lipopeptaibols (6–10 residues) [161]. Well-known representatives of the long-chain peptaibols are alamethicins [162] and trichorzianins [163], while harzianins belong to the short-chain group, and trichogin A to the lipopeptaibols group [164].

The amphipathic nature of peptaibols enables them to form artificial membrane pores, create voltage-dependent ion channels, facilitate cytoplasmic exchange, and cause cell death. Many peptaibols also act as elicitors of plant defense, linking antibiosis with induced systemic resistance (ISR) [47].

**Figure 7:**
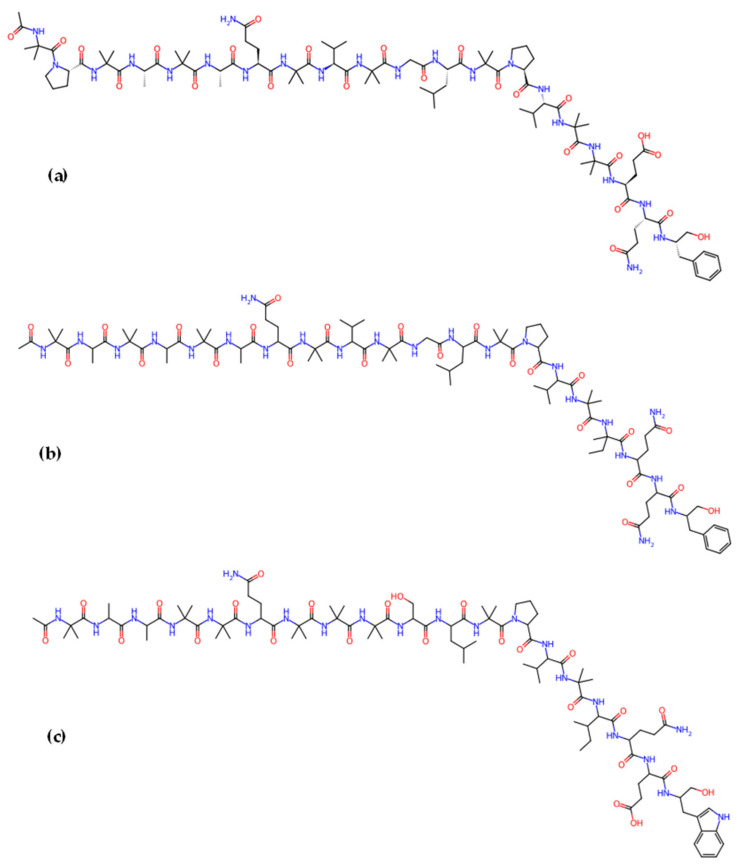
Structure of antifungal secondary metabolites produced by *Trichoderma* spp. (**a**) Alamethicin; (**b**) Triharzianin B; (**c**) Trichokonin VII. Available online at https://pubchem.ncbi.nlm.nih.gov/ (accessed on 25 November 2025).

Alamethicin (Figure 7a) is part of a family of fungal peptaibol antibiotic peptides rich in hydrophobic amino acids that self-assemble when interacting with lipid membranes [165]. It is one of the most studied membrane-active antibiotic peptides from *T. viride*, composed of 20 amino acids, known for forming pores in lipid bilayer membranes and inducing plant systemic resistance [166]. *T. harzianum* is known to produce 55 different peptaibols (subfamilies and groups) with 11-, 14-, 18-, and 19-residue variants.

Triharzianins are a subgroup of 19-residue peptaibols that act synergistically with *T. harzianum* chitinases and β-1,3-glucanases against *B. cinerea* [167]. Triharzianin B, a short-chain peptaibol produced by *T. harzianum*, displays inhibitory activity against *A. fumigatus*, *T. edulis*, and *Tricholoma matsutake* [168]. Nafuredin C, a polyketide derivative isolated from *T. harzianum* D13, shows significant antifungal activity against several phytopathogens, including *B. cinerea*, *Magnaporthe grisea*, *Ph. parasitica*, *Pestalozzia theae*, and *Valsa mali* [169].

Spirosorbicillinol D, a hexaketide-vertinoid produced by *T. longibrachiatum*, demonstrates antifungal activity against *Ph. infestans* [170].

Trichokonins (TKs), a group of small, cyclic peptides, are synthesized by a nonribosomal peptide synthetase (NRPS) pathway. Isolated from *T. pseudokoningii*, they were initially identified by Huang et al. [171]. These compounds demonstrate broad-spectrum antimicrobial activity and high stability, presenting significant potential for application as biological control agents in sustainable crop protection. Isolated and purified TKs from the strain *T. pseudokoningii* SMF2 comprise isoforms such as Trichokonin VI, Trichokonin VII (Figure 7c), and Trichokonin VIII [172]. Trichokonin VI (TK VI) not only promotes growth in moth orchids but also triggers induced systemic resistance against the pathogenic fungus *B. cinerea* [173]. Research indicates that TK VI effectively inhibits pathogenic fungi and bacteria and induces apoptosis in tumor and fungal cells [174,175].

Peptaivirins are special peptaibols isolated from *Trichoderma* spp., rich in Aib, and have an N-terminus of acetylated phenylalanine. Peptaivirin analogs (A and B), which show antiviral activity against the tobacco mosaic virus [160].

Epipolythiodioxopiperazines (ETPs) are a class of biologically active fungal secondary metabolites produced by nonribosomal peptide synthetases. They feature a disulfide-bridged dioxopiperazine ring and are derived from two amino acids. The toxicity of ETPs primarily results from their disulfide bridges, which readily interact with thiol groups on target proteins, leading to protein inactivation and cellular damage [176]. Viridin, gliotoxin, and gliovirin are among the most well-known metabolites associated with antibiosis.

**Figure 8:**
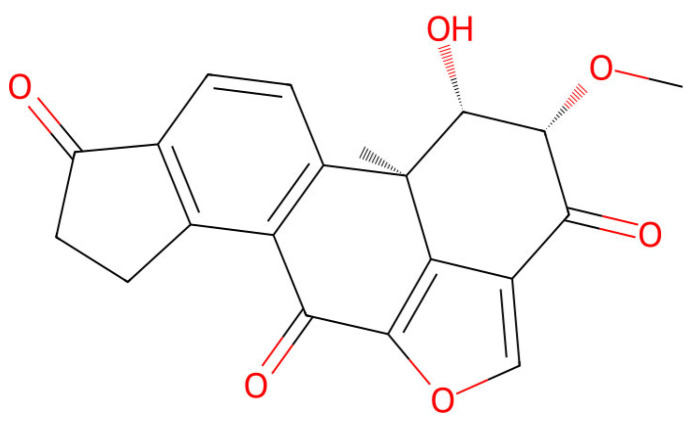
Structural formula of viridin. Available online at https://pubchem.ncbi.nlm.nih.gov/ (accessed on 10 November 2025).

Viridin (Figure 8), first described by Brian and McGowan [177] in 1945, is a furanosteroid antibiotic produced by *T. virens* (formerly *Gliocladium virens*). It is known for its potent antimicrobial properties and activity against *R. solani* [178], *Pythium ultimum*, *Meloidogyne incognita* [179], and *B. cinerea* [180]. It is biosynthesized from a steroid precursor through oxidative reactions catalyzed by cytochrome P450 enzymes. Viridin primarily inhibits serine/threonine protein kinases, including phosphatidylinositol 3-kinase (PI3K) and protein kinase C (PKC), thereby disrupting essential cellular signaling pathways in target pathogens [181].

Gliotoxin, synthesized by an NRPS pathway in several fungi, including *T. virens* and *A. fumigatus*, is a promising biological agent for controlling soil-borne plant diseases. The gliotoxin-producing strain *T. virens* GL 20 was the first biocontrol agent to be commercially developed and marketed as SoilGard, which protects plants against root and crown rot diseases caused by pathogens such as *Pythium* and *Rhizoctonia* [182].

### 4.6. Volatile Organic Compounds (VOCs)

Many bacteria, such as *Bacillus* and *Pseudomonas*, and fungi, including *Trichoderma* spp. and yeasts, release volatile organic compounds (VOCs), such as alcohols, ketones, terpenoids, and sulfur compounds. These airborne substances easily diffuse through soil pores and the plant canopy, inhibiting spore germination and mycelial growth without requiring direct contact. Some VOCs, like 2,3-butanediol, also act as signaling molecules that trigger host plant defenses. The vast diversity of VOCs makes studying their mechanisms difficult, but evidence suggests they disrupt membranes, cause oxidative stress, and interfere with fungal signal transduction. An extensive review of VOCs produced by *Trichoderma* has recently been published by Jiménez-Bremont et al. [183], detailing their role in plant growth and pathogen defense responses.

A focus on the structural diversity, biological activities, and promising biosynthetic potential of terpenoids produced by *Trichoderma* spp. is reported in the literature [184]. For example, lactone 6-pentyl-α-pyrone (6PP) has antibiotic and flavoring properties, exhibits biocontrol activity in vivo, and demonstrates antifungal activity against multiple plant pathogens in vitro [185].

Pyrone 6-PP, produced by *T. viride* [186], *T. koningii* [187], and *T. harzianum* [188], inhibits the growth of *B. cinerea*, *F. oxysporum*, and *R. solani* [189]. Applying pyrone 6-PP reduced postharvest rot in kiwi fruit caused by *B. cinerea* [190]. In another study, Scarselletti and Faull [191] examined the in vitro antifungal activity of 6-pentyl-α-pyrone, a metabolite produced by *T. harzianum*. The study found that this compound inhibited the growth of the plant pathogens *R. solani* and *F. oxysporum* ssp. *lycopersici*. Wang et al. reported the microbial metabolite Cytosporone S, which showed antimicrobial activity against several Gram-positive and Gram-negative bacteria and fungi [192].

The most abundant compounds (VOCs) from *T. atroviride* strains were 3-methyl-1-butanol, 6-pentyl-2-pyrone, 2-methyl-1-propanol, and acetoin, which exhibited potent inhibitory effects on the mycelial growth of *Ph. infestans*, causing morphological and ultrastructural damages [193]. VOCs produced by *T. koningiopsis* T-51 showed high inhibitory activity against plant pathogenic fungi, *B. cinerea* and *F. oxysporum* [194].

Recently, it was shown that VOCs produced by *Pseudomonas* species, especially members of the *P. fluorescens* complex, exhibit significant antifungal activity against a wide range of phytopathogenic fungi [195,196]. *R. solani* was inhibited by VOC ketone compounds 2-nonanone and 2-undecanone emitted by *P. chlororaphis* 449 [197], while *B. cinerea* growth was suppressed by several *P. fluorescens* VOCs, including DMDS, DMTS, geranyl formate, acetic acid, butyric acid, isobutyric acid, 2-methylbutyric acid, and isovaleric acid. The VOCs alcohols 3-methyl-1-butanol, phenylethyl alcohol, and 2-methyl-1-butanol inhibited mycelial growth and spore germination in *Ceratocystis fimbriata* and showed wide-spectrum antifungal activity against several plant pathogenic fungi [198].

## 5. Engineering and Strain Improvement of Biofungicide Producers

### 5.1. Genetic Engineering

The production of biofungicides by *Bacillus* species can be significantly enhanced through targeted genetic engineering and strain improvement strategies, including promoter engineering, deletion and overexpression of key biosynthetic genes, and manipulation of global transcriptional regulators. Such approaches are particularly relevant for non-ribosomal peptide synthetases (NRPSs) and polyketide synthases (PKSs), which are responsible for the biosynthesis of many important lipopeptides and polyketides with antifungal activity, and represent key targets for genetic optimization to increase yield, activity, or specificity of these bioactive compounds.

Significant improvements in the expression of the *bac* operon were achieved in recombinants *B. amyloliquefaciens* FZBREP and FZBSPA by replacing the native promoter with constitutive PrepB and Pspac promoters derived from pMK3 and pLOSS plasmids, respectively. This approach resulted in 2.7- and 4.16-fold increases in bacilysin production compared to the wild-type FZB42. The highest final bacilysin titer was recorded for FZBSPA at 7.73 g/L [199]. Although this study did not test antifungal activity, bacilysin is well known for its potent effect against *Candida albicans*, acting by hydrolysis to anticapsin, which inhibits the aminotransferase activity of glucose-6-phosphate synthase in the fungus [200]. Similarly, *B. subtilis* BBG100, a derivative of strain ATCC 6633, was engineered by replacing the mycosubtilin operon promoter with a constitutive promoter from the *repU* gene of *S. aureus*. This modification resulted in a 15-fold increase in mycosubtilin production, reaching a peak titer of 203 mg/L after 72 h, and the engineered strain showed approximately twice the inhibition zones against *B. cinerea*, *F. oxysporum*, and *Ph. aphanidermatum* compared to the wild type [201].

In another study, the spontaneous mutant strain *B. subtilis* BBG21, which overproduces fengycin at levels 7 to 30 times higher than strains such as ATCC 21332, BBG111, and FZB42 (up to 480 mg/L), served as a source of a strong promoter. The fengycin promoter (Pfen) from BBG21 was cloned into strain BBG111, creating BBG203, which exhibited an eightfold increase in fengycin production. In contrast, using the fengycin promoter from ATCC 21332 did not enhance production, and the maximum fengycin titer achieved by BBG203 was only 11.5 mg/L, highlighting limitations from a biotechnological perspective [202].

Genomic deletions of the iturin and fengycin biosynthetic clusters in *B. amyloliquefaciens* GR167, combined with substitution of the native promoter in the *srf* operon with PRsuc and PRtpxi, increased surfactin production by 10.4-fold, reaching a maximum of 311 mg/L. The promoter replacements were guided by screening 18 endogenous promoters in *B. amyloliquefaciens* LL3, which revealed an unusual lack of correlation between RNA-seq FPKM values and GFP fluorescence used as a reporter [203].

Genetic engineering studies have further clarified the crucial roles of global transcription regulators CodY, ComA, DegU, and Spo0A in the biosynthesis of bacillomycin D, fengycin, and surfactin by *B. amyloliquefaciens* fmbJ. Overexpression of *spo0A* under the *Pgrac* promoter in the pHT43 vector increased production of all three antifungal agents: bacillomycin D increased 2.34-fold, reaching nearly 649 mg/L after 72 h of cultivation with 100 mg/L (0.35 mM) IPTG; fengycin increased 3.2-fold to 245 mg/L; and surfactin increased 1.7-fold. The effects of the other global regulators were more variable. Overexpression of degU increased fengycin production (3.7-fold, to 279 mg/L) but decreased surfactin production (approximately twofold). Overexpression of *comA* moderately increased fengycin and surfactin levels, whereas *codY* had negligible effects. Knockout studies also demonstrated diverse outcomes: deletion of *codY* and *degU* improved surfactin production by about 30% (reaching 7–8 mg/L) but predictably decreased fengycin and bacillomycin D levels, whereas deletion of *comA* or *spo0A* drastically reduced production of all three compounds [204].

These studies highlight the complexity of transcriptional regulation in antifungal compound biosynthesis, emphasizing the importance of careful strain engineering. Surfactin production and sporulation in *Bacillus* species have been extensively studied, often resulting in seemingly conflicting findings. For example, *B. subtilis* JABs32, a sporulation-deficient strain with inactivated *spo0A*, produced an impressive 26 g/L of surfactin after 36 h of fed-batch cultivation, nearly four times more than the sporulating strain JABs24 [205]. Conversely, among non-sporulating mutants created through knockout of *spo0A*, *spoIIIE*, or *spoIVB* in *B. subtilis* TS1726, only the *spo0A*-null strain did not produce surfactin. The *spoIVB*-null mutant showed the highest surfactin yield among the knockouts, reaching 9.6 g/L after 60 h—less than 16% above the parent strain. Production increased further by 74% (16.7 g/L) following supplementation with leucine (5 g/L) and the introduction of genes involved in leucine biosynthesis (*leuABCD*, *ilvK*) [206].

### 5.2. Metabolic Engineering and CRISPR-Based Strategies for Biofungicide Improvement

Metabolic engineering has been studied to increase antifungal compound production in Bacillus species, though improvements are often modest. *B. subtilis* BBG261 was engineered by modifying the metabolic pathway that breaks down branched fatty acids, specifically targeting the *bkd* operon, which includes *lpdV*, *bkdAA*, *bkdAB*, and *bkdB*, responsible for the final step. However, an *lpdV* knockout mutant showed only a 1.6-fold increase in specific surfactin yield (419 mg/g DW) after six hours of growth compared to the parent strain BBG258. Conversely, strain BBG260, created by deleting the global transcription regulator *codY*, produced a 5.7-fold higher specific surfactin yield (1483 mg/g DW) under the same conditions. Notably, BBG260 also achieved a significant increase in surfactin titer, reaching 2289 mg/L after 10 h of growth, which is 10 times higher than that of the parent strain and 9 times higher than that of BBG261. A minor advantage of the *lpdV* mutant was its slightly improved ability to produce the rare isoform with a C14 fatty acid chain. However, this increase was only 12% compared to the *codY* mutant [207].

Similarly, *B. subtilis* BSJ00, a strong fengycin producer (121.20 mg/L) derived from strain 168 through overexpression of *sfp* and *degQ* and cultivated using water-soluble soybean cake powder (WSCP) as a nitrogen source, which proved superior to tryptone and yeast extract, was further improved, albeit slightly, through promoter engineering, achieving only a 13% increase in fengycin titer. A substantially greater enhancement, however, was achieved by increasing the supply of fatty acyl-CoA via deletion of *fadB* and overexpression of *yhfL*, *yngH*, and *tesA*, resulting in a 2.13-fold increase to 258.41 mg/L fengycin [208].

Extensive metabolic engineering was also applied to *B. amyloliquefaciens* WH1 to enhance its antifungal activity. A quadruple knockout of *kinA*, *bdh*, *dhbF*, and *rapA*, aimed at increasing branched amino acid availability and disrupting sporulation, was combined with overexpression of *sfp*, which encodes the enzyme 4-phosphopantetheinyl transferase essential for the CoA activation step in lipopeptide biosynthesis. The engineered strain produced significantly higher iturin and fengycin titers, reaching 31.1 mg/L and 175.3 mg/L in flask fermentation, and 123.5 mg/L and 1200.8 mg/L in a 50 L bioreactor, respectively. Compared to the parent strain, which produced 5.4 mg/L iturin and 75.2 mg/L fengycin, the total production of fengycin and iturin increased by 16- and 23-fold, respectively [209].

**Figure 9:**
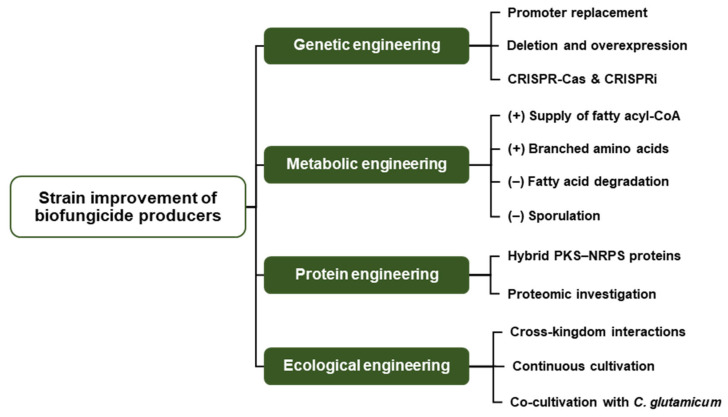
Schematic overview of the methods to increase the antifungal activity of microbial strains: targeted genetic and metabolic engineering and other strain improvement strategies.

Genome editing using CRISPR-Cas9 has also been employed to improve secondary metabolite production in *B. subtilis* (Figure 9). For example, the system was used to produce amorphadiene, a precursor to artemisinin, in *B. subtilis* 168; however, the improvement was modest, with less than a 50% increase in titer from 81 to 116 mg/L after 48 h of flask fermentation [210]. Artemisinin, derived from *Artemisia annua* (sweet wormwood), is an important antimalarial drug, and extracts from this plant possess strong antifungal properties [211].

CRISPR interference (CRISPRi) has been successfully used to enhance surfactin production in various *B. subtilis* 168 derivatives. Targeted repression of 16 out of 20 genes led to significant increases in surfactin levels, with notable 3.18-, 2.47-, and 2.41-fold improvements for *yrpC* (0.54 g/L), *murC* (0.42 g/L), and *racE* (0.41 g/L), respectively, compared to strain BS168NU-Sd (0.17 g/L), which expresses dCas9 without the single guide RNA (sgRNA). The simultaneous silencing of *yrpC* and *racE*, both involved in L-glutamate metabolism, resulted in a 4.41-fold increase in surfactin titer (0.75 g/L). Interestingly, the strain lacking dCas9 expression (BS168NU-S) achieved a higher surfactin titer (0.37 g/L), which the authors attributed to the toxic effects of dCas9 expression.

### 5.3. Protein Engineering

Hybrid PKS–NRPS proteins, created by fusing fungal polyketide synthases (PKSs) with nonribosomal peptide synthetases (NRPSs), have attracted significant interest as a means to expand the diversity of fungal secondary metabolites with potential antifungal effects. One study examined 57 fusion constructs made through yeast-based recombination, linked to the biosynthetic pathways of cyclopiazonic acid, lovastatin, and pseurotin [212]. The latter two compounds show fungicidal activity against zygomycetes [213] and fungistatic activity against *C. albicans* [214]. Rational engineering and module swapping of PKS–NRPS hybrids from *A. nidulans* led to the creation of two new pre-cytochalasin intermediates, niduclavin and niduporthin [215]. Although their antifungal properties were not tested, cytochalasins are part of the broader cytochalasan family, characterized by a tricyclic core and known to have some antifungal activity, though not widespread or particularly strong [216].

Proteomics, although not directly used in hybrid PKS–NRPS engineering, remains a valuable method for discovering strategies to improve strain performance. A proteomic analysis of *B. amyloliquefaciens* X030 identified a core proteome of 1160 proteins (>50% of the total) measurable at 10, 24, and 34 h of cultivation. The most abundantly expressed proteins at different time points included several involved in bacillomycin Lb biosynthesis (FabG, DapG, AroA, Rpe, PdhD, YhdR), potential regulatory proteins (PerP, PhoP, CcpA, CsfB), and enzymes involved in fatty acid biosynthesis, glycolysis, the TCA cycle, and, most notably, the metabolism of amino acids such as serine, aspartate, glutamate, and tyrosine [217].

A proteomic investigation of *B. velezensis* ES1-02, a lipopeptide-producing strain active against the phytopathogenic fungus *Diaporthe* spp., identified 148 differentially expressed proteins (66 upregulated, 82 downregulated) in the presence of *D. longicolla* DPC_HOH20. Nearly one quarter (23%) of the upregulated proteins were associated with the biosynthesis of bioactive secondary metabolites, including bacillaenes and polyketides. Interestingly, *B. velezensis* ES1-02 produced tenfold higher levels of surfactin compared with its standard yield (97.4 mg/L) when co-cultured with *D. longicolla* DPC_HOH20. However, the authors only presented HPLC chromatograms to support this pronounced effect; surfactin titers in the co-cultures were not reported [218].

### 5.4. Ecological Engineering and Cross-Kingdom Modulation of Antifungal Metabolite Production

Cross-kingdom interactions between fungi and bacteria are increasingly used to improve the performance of antifungal metabolite producers. Cooperative behavior between *B. velezensis* and *T. guizhouense*, two beneficial but antagonistic species, enhanced resistance to *Fusarium* wilt disease (FWD) in tomatoes. A major facilitator superfamily transporter, TgMFS4, in *T. guizhouense* was identified as crucial for cross-kingdom communication with *B. velezensis*. Deletion of the corresponding gene (*tgmsf4*) reduced bacilysin uptake by *T. guizhouense* and weakened the synergistic antagonism. Inoculating tomato plants with the *Δtgmsf4* mutant and *B. velezensis* still increased resistance to FWD compared to inoculation with the wild-type strain [219].

Continuous cultivation of *B. subtilis* BBG116, a constitutive mycosubtilin overproducer, in a foam-overflow bioreactor enabled in situ recovery of over 99% of the product and led to a twofold increase in mycosubtilin productivity (1.18 mg/g DW/h), surpassing previous reports [220].

In *T. harzianum*, the C6 zinc finger protein Thc6, a key regulator of induced systemic resistance (ISR) against *Curvularia* leaf spot in maize, was shown to control the expression of two hydrolases, Thph1 and Thph2. These proteins regulate ROS and calcium homeostasis, and knockout mutants, along with RT-qPCR evidence, confirmed their importance for effective root colonization and the potential activation of ISR in maize [221].

Overexpression of genes involved in proline uptake (*opuE*, *putP*, *gabP*) in *B. subtilis* GGF26 led to modest improvements (<16%) in fengycin production after 72 h of fermentation (872 mg/L compared to 753 mg/L in the control). These gains were only observed when 8 g/L of proline was added. Overexpressing transporters for isoleucine, alanine, and threonine resulted in increases of 47%, 36%, and 8%, respectively, with the highest absolute titer (942 mg/L) achieved for threonine supplementation. Notably, coculturing with the high-proline-producing *Corynebacterium glutamicum* yielded 1555 mg/L of fengycin in shake flasks—double the amount produced in monoculture—and reached an impressive 2310 mg/L in a 5 L bioreactor after 96 h, representing a nearly 49% increase [222] (Table 4).

A spontaneous mutant of *T. guizhouense*, obtained during protoplast transformation of strain NJAU 4742, was found to overproduce harzianic acid (HA) and related derivatives. The mutant showed significantly higher antifungal activity against *Neurospora crassa*, *Alternaria alternata*, *B. cinerea*, and *F. odoratissimum*, as well as against multiple *Trichoderma* species (*T. afroharzianum*, *T. reesei*, and *T. virens*). Metabolomic, bioinformatic, and evolutionary analyses of the HA biosynthetic gene cluster (*hacBGC*) identified two transcription factors, HacI and HacF, that positively regulate HA biosynthesis and its export [224].

As in *Bacillus* spp., regulatory network modifications in *Trichoderma* can lead to contradictory or unintended outcomes. In *T. harzianum*, disruption of *hog1*, a homolog of the yeast MAPK HOG1, conferred improved resistance to osmotic stress but simultaneously reduced antifungal activity against *Phoma betae* and *Colletotrichum acutatum*. Hog1 appeared to play only a minor role in oxidative stress responses, emphasizing the interconnected nature of fungal signaling pathways [225]. Overexpression of the transcriptional coactivator MBF1 in *T. harzianum* T34 negatively impacted antifungal activity against *F. oxysporum* and *B. cinerea*. Furthermore, deletion of *ctf1*, which encodes another transcription factor, resulted in loss of yellow pigmentation and elimination of 6-pentyl-2H-pyran-2-one and related antifungal volatiles [226]. The transcription factor PacC, a central regulator of the Pal/Rim pH-signaling pathway, was required for homodimericin A production in *T. harzianum* 3.9236 [227]. However, its influence on antifungal activity was limited. PacC mutants showed little to no change in antagonism toward *F. fujikuroi* and *R. solani*. Only a slight improvement was observed against *Sclerotinia sclerotiorum* [228].

## 6. Conclusions

This review highlights the primary factors driving the global increase in biofungicide use, as well as the key challenges that still hinder widespread adoption. Consumer expectations for sustainable, residue-free agricultural products and increasingly strict regulatory frameworks make biofungicides essential for modern pest management. Their environmental friendliness makes them attractive for organic green farming. Flexible regulations, diversified formulations with improved stability, and easier application methods also facilitate the development of the biofungicide industry. Moreover, the advances in biotechnology have led to more powerful and targeted biofungicides, including systemic types that offer longer-lasting protection and reduce application frequency. Improvements in shelf life for liquid and freeze-dried products have expanded distribution options. At the same time, innovations in delivery methods, such as incorporation into irrigation systems and increased drone use, have also occurred.

Despite all these positive developments, several challenges remain for the global adoption of biofungicides. Their effectiveness heavily depends on environmental conditions, and they perform poorly in humid tropical regions. Production and application costs are 20–30% higher than those of chemical fungicides, and many formulations still require low-temperature storage, which adds logistical hurdles. Regulatory approval processes, although improving, remain lengthy and complex, and high R&D costs limit participation by smaller companies.

In conclusion, innovation, environmental pressure, and rising market demand are fueling the rapid growth of biotechnologically enhanced biofungicide production. Unlocking these products’ full potential, however, will require coordinated advances in strain engineering, formulation stability, cost-effective manufacturing, regulatory harmonization, and farmer education. Collectively, these factors will determine how quickly biofungicides transition from a promising alternative to a primary standard in sustainable crop protection.

## Figures and Tables

**Table 1:** Microbial and Plant-Derived Antifungal Agents Used in Biological Control.

Strain/Plant	Antifungal Agent/Producer	Effect on Fungal Pathogens/Mode of Action	Reference
*Bacillus* spp. (PGPR)	*B. subtilis*, *B. amyloliquefaciens*, *B. licheniformis*, *B. cereus*	Production of lipopeptides, polyketides, antimicrobial peptides, siderophores; suppression of *Fusarium*, *R. solani*, *Macrophomina*, *Alternaria*, *Penicillium* spp.	[30,31]
*B. amyloliquefaciens*	FZB42: Bacillomycin D	Inhibition of *Fusarium graminearum* growth	[35]
*B. subtilis* BS155	Fengycin	Disruption of fungal cell membrane integrity, oxidative stress, and hyphal death (*Magnaporthe grisea*)	[35]
*B. siamensis*	Iturins, bacillomycin F; chitinase, β-1,3-glucanase	Cell wall degradation and enhanced suppression of *Colletotrichum*, *R. solani*, *M. grisea*, *Fusarium* wilt	[36]
*B. velezensis* SDTB038	Bacillaene, bacilysin, difficidin, fengycin, macrolactin, surfactin	Combined metabolite action controlling *Fusarium* crown and root rot	[37]
*Ps. piscium* ZJU60	Phenazine-1-carboxamide	Reduced virulence and mycotoxin production in *F. graminearum*	[38]
*Ps. aeruginosa*	Multiple metabolites + ISR induction	Suppression of *Colletotrichum capsici* and induction of plant systemic resistance	[39]
*Streptomyces* spp.	Diverse secondary metabolites	Inhibition of *Fusarium*, *Rhizoctonia*, *Botrytis*, *Alternaria*, *Ganoderma*, *Phytophthora* spp.; enhanced via fermentation optimization and genetic engineering	[41,42]
Yeasts	*Candida oleophila*, *Hanseniaspora anomala*	Competition, rapid colonization, suppression of *P. digitatum*, *P. italicum*, *Geotrichum candidum* (up to 100% disease control)	[43]
Yeasts + *Bacillus* spp.	*C. oleophila*, *Debaryomyces hansenii*, *Bacillus* spp.	Biofilm formation, lytic enzymes, lipopeptides, volatile compounds; control of green and blue mold during storage	[44]
*Trichoderma* spp.	*T. harzianum*, *T. viride*, *T. atroviride*, *T. hamatum*, *T. asperellum* ICC012, *T. gamsii* ICC080	Cell wall degradation via chitinases, glucanases, and proteases; broad-spectrum pathogen inhibition. Endophytic colonization, upregulation of defense genes, and reduction in *Fusarium* head blight	[45,46]
Arbuscular mycorrhizal fungi	*Funneliformis mosseae* + *Sinorhizobium medicae*	Indirect biocontrol via nutrient uptake and induced systemic resistance; suppression of *F. oxysporum*	[48]
Plant secondary metabolites	Terpenoids, phenolics, alkaloids, flavonoids	Inhibition of spore germination, DNA/protein synthesis, hyphal damage, and mycotoxin reduction	[50,51]
Plant essential oils	*Thymus*, *Origanum*, *Rosmarinus*, *Mentha*, *Ocimum*, *Reynoutria sachalinensis,* citrus	Membrane disruption and growth inhibition of *Botrytis*, *Fusarium*, *Alternaria*, and *Penicillium* spp.	[52]
Bryophyte extracts	*Porella*, *Cinclidotus*, *Anomodon*	Inhibition of *Botrytis cinerea* mycelial growth	[53]
Angiosperm extracts	*Ipomoea batatas*, *Myristica fragrans*, *Curcuma longa*	Ergosterol biosynthesis disruption, membrane damage, antifungal and antioomycete effects	[55,56,57]
Phenolic-rich plant extracts	Rice straw, mistletoe, *Cinnamomum camphora*	Increased membrane permeability, cytoplasmic leakage, and induced resistance	[58,59,60,61,62]
Medicinal plant extracts	*Eryngium campestre*, *Argyranthemum frutescens*	Dose-dependent inhibition of fungal growth via polyphenols and polyacetylenes	[61,64]

**Table 2:** Bacterial production of secondary metabolites with antifungal properties.

Species	Antifungal Metabolite	Gene Clusters/ Domains	Cluster Regulation	Target Pathogens/Crops	Reference
*B. subtilis*	Iturin ^1^	*ituA*–*D* NRPS modules with adenylation (A), condensation (C), thiolation (T) domains	Controlled by Spo0A and DegU, the nutrient limitation enhances expression	*Fusarium* spp., *B. cinerea*, *R.* spp./cereals, vegetables	[127]
*B. subtilis*	Fengycins ^1^	*fenA*–*E*; multi-modular NRPS genes encoding β-hydroxy fatty acid linkage	Co-regulated with surfactin cluster; stress-responsive	*Rhizopus stolonifer*, *Alternaria alternata*	[128,129]
*B. subtilis*	Surfactin ^1^	*srfAA*, *srfAB*, *srfAC* NRPS	Linked to competence/sporulation via ComA and Spo0A	Synergistic with iturins/fengycins; biofilm suppression	[130]
*S. griseus*	Candicidin ^2^	*canP1*–*canP6*; modular type I PKS; tailoring genes for glycosyltransferase and oxidoreductase	Cluster-specific regulators (canR); silent under lab conditions unless activated	*Candida* spp., *Fusarium* spp.	[131]
*S. nodosus*	Amphotericin B ^2^	*amphA*–*C*; modular type I PKS enzymes, tailoring genes for Cytochrome P450 monooxygenase, glucosyltransferase	regulated by the cluster-specific activator amphR, stress-responsive regulators (PhoP/PhoR, AdpA, ppGpp)	*Aspergillus*, *Candida*, *Cryptococcus*	[132]
*S. noursei*	Nystatin ^2^	*nysA*–*nysH*; modular type I PKS enzymes; tailoring enzymes for glycosylation	Regulated by pathway-specific transcription factors (nysRI–RIV)	Broad antifungal activity; model for macrolide biosynthesis	[133]
*S. natalensis*	Natamycin ^2^	*pimS0*–*pimS4*; type I PKS tailoring enzymes, transport, and regulation glycosylation	Regulated by pathway-specific regulators PimM, PimR, and PimT	Broad antifungal activity; food industry	[134]
*P. syringae * pv. *syringae*	Syringomycin ^1^	*syrB1*, *syrB2*, *syrC*, *syrE* NRPS modules (A–T–C); halogenase SyrB2	GacS/GacA, SalA, iron- responsive regulation	*A. flavus*, *A. niger*, *A. fumigatus*, *F. moniliforme*, *F. oxysporum*	[135,136]
*P. syringae * pv. *syringae*	Syringopeptin ^1^	*sypA*, *sypB*, *sypC* (≈22 NRPS modules)	Co-regulated with syr cluster; syr/syp promoter box; GacS/GacA	*R. solani*, *Fusarium* spp., *Pythium* spp., *Phytophthora* spp., *B. cinerea*, *Verticillium* spp.	[121,137]
*P. syringae*	Syringofactins ^1^	*sfaA*, *sfaB*, *sfaC*, *syfA*, *syfB*	GacS/GacA; plant surface induction	Promote leaf-surface (epiphytic) colonization	[138]
*P. fluorescens* SBW25/SS101	Viscosin ^1^	*viscA*, *viscB*, *viscC*; NRPS	GacS/GacA, quorum sensing	*Rh. solani*, *Alternaria* sp., *F. oxysporum*, *Pythium debaryanum*	[139,140]
*P. putida* PCL1445	Putisolvins ^1^	*psoA*, *psoB*, *psoC*	Quorum sensing (PsoR); surface-associated induction	*F. oxysporum*	[141]
*P. fluorescens* Pf-5/SS101	Massetolides ^1^	*masA*, *masB*, *masC*	GacS/GacA; RpoS; plant root–dependent cues	*Alternaria* sp., *F. oxysporum*	[142]
*P. protegens* Pf-5	Orfamides ^1^	*ofaA*, *ofaB*, *ofaC*	GacS/GacA; RsmA/RsmE post-transcriptional regulation	*Magnaporthe* *oryzae*	[143]
*P. fluorescens*	Arthrofactin ^1^	*arfA*, *arfB*, *arfC*	QS-related control; surface-motility signals	*Fusarium* spp., *Aspergillus* spp., *B. cinerea*	[144]
*Pseudomonas* sp.	Entolysins ^1^	*etlA*, *etlB*, *etlC*	GacS/GacA; environmental regulation	*Rh. solani, Colletotrichum* spp.	[145]

^1^ Cyclic lipopeptides, ^2^ Polyene macrolides.

**Table 3:** Production of secondary metabolites with antifungal activity by *Trichoderma* spp.

Species	Antifungal Metabolite	Gene Clusters/Domains	Cluster Regulation	Target Phytopathogens	Reference
*T. koningii*	Trichokonins VI; VII; VIII	NRPS cluster; multiple A-T-C modules	Environmental stress, nutrient limitation, and antagonistic interaction with pathogens	*B. cinerea*, *R. solani; F. oxysporum*, *Sclerotinia sclerotiorum*	[156]
*T. harzianum*	Peptaibols ^1^	*tex*1 type NRPS with repeated A-T-C modules incorporating α-amino isobutyric acid	Induced by host hyphal contact; regulated by MAPK ^2^ pathways	*F. oxysporum*, *Alternaria alternata*	[157]
*T. virens*	Polyketides ^3^	*pks*4, *gliP*-like genes, PKS, and hybrid NRPS–PKS clusters	Controlled by LaeA/Velvet complex, responsive to carbon limitation	*Sclerotinia sclerotiorum*, *Botrytis* spp.	[158]
*T. virens*	Peptaivirin A/B ^4^	*pivA/pivB* NRPS gene with A-T-C-TE domain organization	Modulated by signaling pathways associated with biocontrol activity	*R. solani*, *F. oxysporum* *B. cinerea*, *Ph. infestans*	[159,160]
*T. harzianum*	Trichorzianine A1/B1 ^4^	*trz* NRPS gene; contains A, T, C, and TE domains	Expression correlated with conidiation and mycoparasitism	*R. solani*, *F. graminearum* *B. cinerea*	[47]

^1^ Linear NRPSs; ^2^ MAPK, mitogen-activated protein kinase; ^3^ Viridin, gliotoxin-like compounds; ^4^ Peptaibol.

**Table 4:** Genetic engineering approaches to boost microbial antifungal biosynthesis.

Strain	Target	Method	Effect	Highest Titer	Reference
*B. amyloliquefaciens* FZBSPA	Bacilysin	Promoter replacement	3.16-fold higher production	7.73 g/L (48 h)	[199]
*B. subtilis* BBG100	Mycosubtilin	Promoter replacement	15-fold higher production	203 mg/L (72 h)	[201]
*B. subtilis* BBG203	Fengycin	Promoter replacement	8-fold higher expression	11.5 mg/L (48 h)	[202]
*B. amyloliquefaciens* GR167	Surfactin	Deletion of iturin and fengycin clusters; promoter replacement	10.4-fold higher production	311 mg/L (48 h)	[203]
*B. amyloliquefaciens* fmbJ	Bacillomycin D Fengycin	Overexpression of *spo0A* Overexpression of *degU*	2.34-fold higher production 3.7-fold higher production	649 mg/L (72 h) 279 mg/L (72 h)	[204]
*B. subtilis* BBG260	Surfactin	Deletion of *codY*	5.77-fold higher specific yield 10.36-fold higher production	1483 mg/g DW (6 h) 2289 mg/L (10 h)	[207]
*B. subtilis* JABs32	Surfactin	Inactivation of *spo0A*	4-fold higher production	23.7 g/L (31 h)	[205]
*B. subtilis* 168	Surfactin	Knockout of *spo0A* or *spoIVB*	No production in Δ*spo0A*, 15.7% increase in Δ*spoIVB*	9.6 g/L (60 h)	[206]
*B. subtilis* 168	Amorphadiene	CRISPR-Cas9 editing	>50% increased production	116 mg/L (48 h)	[210]
*B. subtilis* H1	Surfactin	CRISPRi silencing of *yrpC* and *racE*	4.41-fold increase	752 mg/L (24 h)	[223]
*B. subtilis* 168	Surfactin	Overexpression of *leuABCD* and *ilvK* Leu supplementation	74% higher production in the Δ*spoIVB* mutant	16.7 g/L (48 h)	[206]
*B. subtilis* BBG261	Surfactin	Knockout of *lpdV*	1.6-fold higher production	252 mg/L (10 h)	[207]
*B. subtilis* BSJ023	Fengycin	Nitrogen source optimized, enhanced supply of fatty acyl-CoA	2.13-fold higher production	258.41 mg/L (48 h)	[208]
*B. amyloliquefaciens* WH1 *B. amyloliquefaciens* WH1	Fengycin	Deletion of *kinA*, *bdh*, *dhbF*, *rapA*; overexpression of *sfp*	2.3-fold increase (flask) 16-fold increase (bioreactor)	175.3 mg/L (48 h) 1200.8 mg/L (48 h)	[209]
	Iturin		5.8-fold increase (flask) 23-fold increase (bioreactor)	31.1 mg/L (48 h) 123.5 mg/L (48 h)	[209]
*B. subtilis* GGF26	Fengycin	Overexpression of Ile, Ala, Pro, and Thr transporters; Co-culture with *Corynebacterium glutamicum* (0.2/0.4 ratio)	47, 36, 16, and 8% higher production 2-fold increase (flask) 49% increase (bioreactor)	872 mg/L (Pro) 942 mg/L (Thr) 1555 mg/L, 72 h 2310 mg/L, 96 h	[222]
*B. velezensis* *T. guizhouense*	*Fusarium* wilt disease	Deletion of *tgmfs4*	Enhanced resistance to *Fusarium* wilt disease	-	[219]
*B. subtilis* BBG116	Mycosubtilin	Overflowing continuous culture in a bioreactor	Continuous recovery >99% 2-fold higher production rate	-	[220]
*T.* *harzianum*	IRS in maize	Knockout of *thph1* and *thph2*	Increased susceptibility to *Curvularia* leaf disease	-	[221]
*B. velezensis* ES1-02	Surfactin	Co-incubation with *Diaporthe longicolla*	10-fold higher production	-	[218]

## Data Availability

No new data were created or analyzed in this study. Data sharing is not applicable to this article.
